# Cap-specific, terminal *N*^6^-methylation by a mammalian m^6^Am methyltransferase

**DOI:** 10.1038/s41422-018-0117-4

**Published:** 2018-11-28

**Authors:** Hanxiao Sun, Meiling Zhang, Kai Li, Dongsheng Bai, Chengqi Yi

**Affiliations:** 1 State Key Laboratory of Protein and Plant Gene Research, School of Life Sciences, Peking University, Beijing, 100871 China; 2 Academy for Advanced Interdisciplinary Studies, Peking University, Beijing, 100871 China; 3 Peking-Tsinghua Center for Life Sciences, Peking University, Beijing, China; 4 Department of Chemical Biology and Synthetic and Functional Biomolecules Center, College of Chemistry and Molecular Engineering, Peking University, Beijing, 100871 China

**Keywords:** Epigenetics, Transcription

Dear Editor,

Dynamic and reversible *N*^6^-methyladenosine (m^6^A) RNA methylation has been found to greatly impact gene expression, leading to the field of epitranscriptomics.^1^ Unlike m^6^A that is an internal modification, a terminal modification at mRNA cap in higher eukaryotes exists, termed as *N*^6^,2′-O-dimethyladenosine (m^6^Am) (Fig. 1a). The first and sometimes the second nucleotide after the *N*^7^-methylguanosine (m^7^G) cap can be methylated at the 2′-hydroxyl group; and when the first nucleotide is 2′-O-methyladenosine (Am), it can be further methylated at the *N*^6^ position to become m^6^Am. m^6^Am was first identified in animal cells and virus mRNA in 1975^2^; several years later the methyltransferase was partially purified and was proposed to be a species whose molecular weight is ~65 KD.^3^ Only very recently, m^6^Am was found to be reversible as well: the first m^6^A demethylase FTO also catalyzed the demethylation of m^6^Am, depending on its sub-cellular localizations.^4,5^ By changing FTO levels, m^6^Am at mRNA cap was also suggested to impair DCP2-mediated mRNA decapping.^4^ However, the methyltransferase of m^6^Am is not unambiguously identified, significantly hindering the functional and mechanistic study of m^6^Am.

**Fig. 1:**
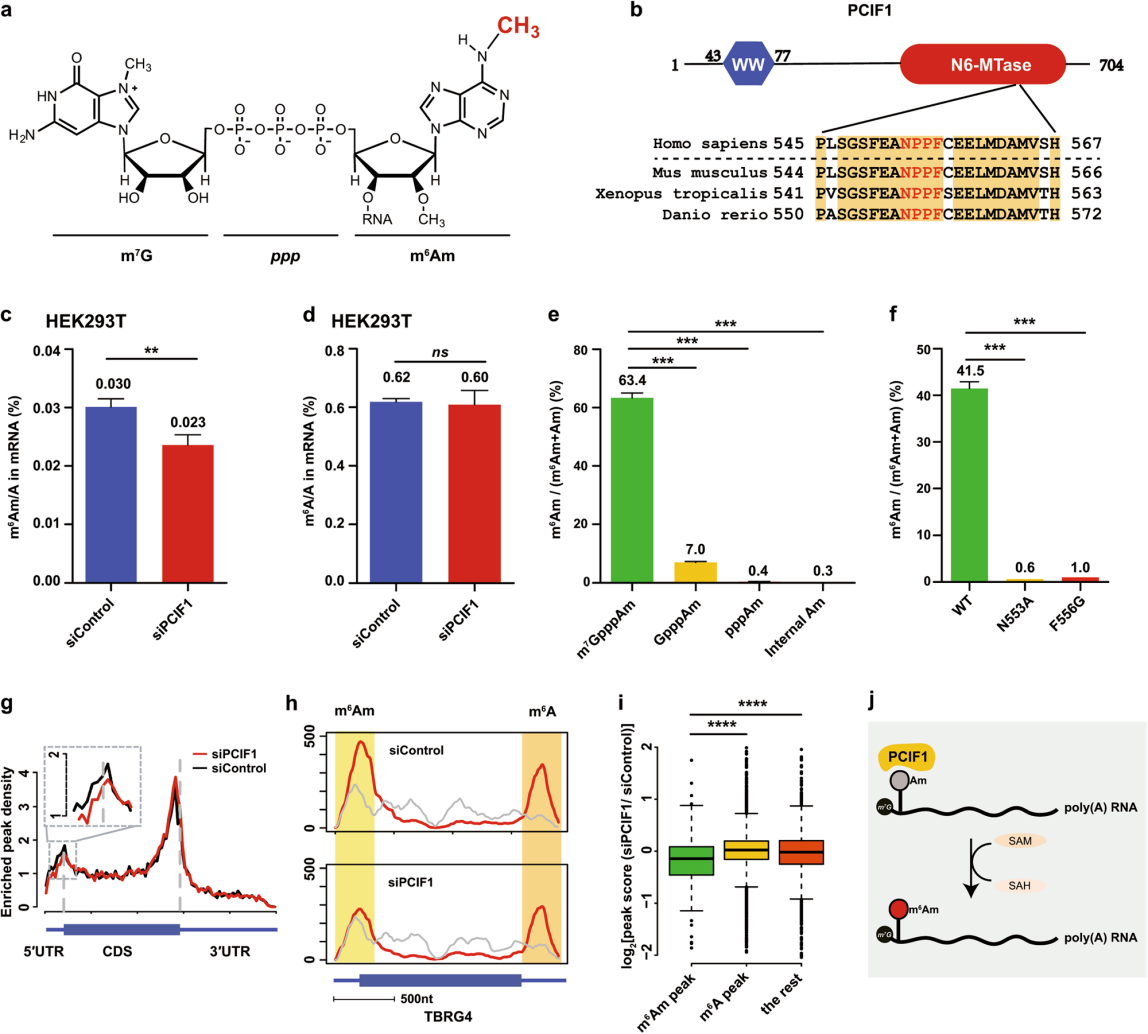
Identification of *N*^6^,2′-O-dimethyladenosine methyltransferase. **a** Chemical structure of m^6^Am, which is adjacent to the m^7^G mRNA cap. **b** Cartoon view of the predicted domain structure of PCIF1, with the conserved “NPPF” motif in the zoom-in view. A sequence alignment is shown below to highlight the high conservation of the key residues for PCIF1 orthologues. Residue 43–77 (blue segment) represents the WW domain, and the red segment denotes the putative catalytic methyltransferase domain. **c** LC-MS/MS quantification of the m^6^Am/A ratios of HEK293T polyA + RNA treated with control or PCIF1 siRNA (*n* = 3). **d** LC-MS/MS quantification of the m^6^A/A ratios of HEK293T polyA+ RNA treated with control or PCIF1 siRNA (*n* = 3). **e** Quantification of the m^6^Am/(Am + m^6^Am) ratios in RNA probes starting with different cap structure (*n* = 3). **f** Quantification of the methylation activity of WT and mutant PCIF1 proteins (*n* = 3). **g** Distribution of enriched m^6^A/m^6^Am peak density across mRNA segments of control and PCIF1 knockdown samples using an m^6^A-seq protocol with random priming. Each segment was normalized according to its average length in Ref-seq annotation. **h** One representative transcript harboring m^6^Am and m^6^A peaks. The m^6^Am peak at the 5′-terminal is significantly decreased upon PCIF1 knockdown, while the m^6^A peak at the 3′-UTR stays the same. The grey line denotes “Input”, and the red line denotes “IP”. **i** Boxplot of log_2_fold change of peak score in PCIF1 knockdown and control mRNA. Enriched peaks are classified into three groups: m^6^Am peak (near TSS and without GGACH motif), m^6^A peak (not in TSS and with GGACH motif), and the rest (potentially m^6^Am + m^6^A). **j** A proposed model for mammalian mRNA m^6^Am modification mediated by PCIF1. *P < 0.05; **P < 0.01; ***P < 0.001; ns, not significant

To clearly identify the methyltransferase, we fractioned the cell lysates of HEK293 cells, which contain robust *N*^6^-methylation activity (Supplementary information, Fig.S1a). This activity was assayed by incubating the column fractions with a 25 nt, synthetic vaccinia virus RNA probe (Probe-1, see Supplementary information) that begins with m^7^GpppAm. We modified the purification route of cell lysates, based on the procedure originally reported^3^ (Supplementary information, Fig.S1b), and subjected the fractions of high *N*^6^-methylation activity to protein identification by sensitive mass spectrometry. We then searched for proteins with putative methyltransferase domain or sequence motif in the list of more than 100 proteins detected by MS, and found a protein named “phosphorylated CTD-interacting factor 1” (or PCIF1) (Fig. 1b; Supplementary information, Fig.S1c), which was bioinformatically proposed to be a DNA/RNA *N*^6^-adenosine methyltransferase.^6^ PCIF1 was originally identified and named due to its ability to directly bind to the phosphorylated C-terminal domain of RNA polymerase II via its WW domain^7^; hence it was speculated to play a role in mRNA biogenesis. However, no enzymatic activity has been reported for PCIF1.

To test whether PCIF1 possesses methyltransferase activity in vivo, we first knocked down *PCIF1* in HEK293 cells by two independent siRNAs and confirmed the knockdown efficiency by qRT-PCR (Supplementary information, Fig.S2a). We then measured the level of m^6^Am in polyA + RNA fraction after decapping using LC-MS/MS. We were able to observe a reduction of m^6^Am level upon *PCIF1* knockdown (Fig. 1c; Supplementary information, Fig.S2b); importantly, the level of the internal m^6^A modification remained unchanged (Fig. 1d), suggesting that PCIF1 is a specific methyltransferase for the terminal m^6^Am. Encouraged by the in vivo results, we then expressed and purified recombinant PCIF1 protein, and tested whether the single protein is capable of methylating RNA substrates under in vitro conditions (Supplementary information, Fig.S2c). The highest activity of PCIF1 was obtained with RNA Probe-1 beginning with a complete cap structure m^7^GpppAm; much lower activity was found with RNA beginning with GpppAm; and barely detectable activity was found with RNA beginning with pppAm or RNA Probe-2 with an internal Am (Fig. 1e). The above enzymatic preference was also supported by biochemical experiments using two different RNA probes (Probe-3 and Probe-4), which in addition showed that the ribose 2′-O-methylation is required for optimal methylation activity as well (Supplementary information, Fig.S2d). Moreover, we introduced point mutations in the highly conserved “NPPF” motif that is characteristic of adenosine methyltransferases, and found that the disruption of this motif reduced the methyltransferase activity of the mutant proteins (Fig. 1f; Supplementary information, Fig.S1d). Because PCIF1 is highly conserved among different species (Fig. 1b), we further tested whether the mouse PCIF1 protein is also functional. We knocked down *mPcif1* by siRNA in mouse NIH-3T3 cells and also observed reduced m^6^Am level (Supplementary information, Fig.S3a, b). Additionally, mouse PCIF1 protein also exhibited a robust methylation activity in vitro (Supplementary information, Fig.S3c). Altogether, the evidence presented above demonstrated that PCIF1 is a novel mammalian m^6^Am writer, which is specific for the 5′-end capped RNA.

To identify the RNA targets of PCIF1, we performed m^6^A-seq experiments for *PCIF1* knockdown and control cells using an anti-m^6^A antibody.^8,9^ Because the antibody recognizes m^6^Am and m^6^A, both types of modifications were enriched and hence detected simultaneously.^10^ m^6^A modifications are known to be enriched around 3′-UTR, with a small portion also present internally in the 5′-UTR; while m^6^Am modifications localized at the 5′-end of RNA. We envisioned that the cap-specific PCIF1 should selectively alter the m^6^Am modification at the 5′-terminal region of transcripts. Indeed, we observed a reduction of modification peaks at the 5′-end but not the 3′-UTR regions of mRNAs upon *PCIF1* knockdown (Fig. 1g; Supplementary information, Fig.S3d). One example is the TBRG4 transcript, for which we found a 5′-end peak and a 3′-UTR peak by m^6^A-seq (Fig. 1h); only the former peak underwent a clear reduction while the latter remained the same. We then grouped the enriched peaks into three categories and again observed significantly decreased signals for the m^6^Am peaks after *PCIF1* knockdown when comparing to the m^6^A and m^6^A + m^6^Am categories (Fig. 1i; Supplementary information, Table S1). We further adopted a different m^6^A-seq procedure that can preserve the 5′-end information of polyA+ RNA, and again found a decrease of m^6^Am peak intensity after* PCIF1* knockdown (Supplementary information, Fig.S3e). In addition, a motif analysis revealed that m^6^Am modification occurs at the transcription start sites, in accordance with the known m^6^Am pattern (Supplementary information, Fig.S3f).^4^ Thus, results from our m^6^A-seq experiments revealed the direct mRNA targets of PCIF1 inside of human cells (Fig. 1j).

Taken together, in this study we revealed the exact identity of the m^6^Am writer protein, characterized its biochemical property and substrate preference, and profiled its cellular targets using an epitranscriptomic sequencing approach. PCIF1 recognizes the positively charged cap structure m^7^GpppAm for optimal activity and is a “stand-alone” RNA methyltransferase. In comparison, the internal m^6^A is installed by a methyltransferase complex, the core components of which are composed of METTL3, METTL14 and WTAP. The m^6^A methyltransferase complex also recognizes internal adenosines, with a preference for those located within a RRACH consensus motif. Hence, while m^6^Am and m^6^A share a common eraser protein FTO, the writer proteins for the two modifications are orthogonal. Manipulating the protein levels of the writers could potentially separate the differential roles of FTO in demethylating m^6^Am and m^6^A. The functional study of m^6^A is greatly facilitated by the discovery and characterization of its regulation system involving the writer, reader and eraser proteins; we envision that the identification of PCIF1 as the m^6^Am writer will pave the path toward functional and mechanistic dissection of this dynamic and reversible epitranscriptomic mark in the future.

## Electronic supplementary material

Supplementary information, figures

Supplementary information, Table S1
