# Supplementary material for: Cap-specific, terminal N6-methylation by a mammalian m6Am methyltransferase
Source: Cell Res. 2018 Nov 28;29(1):80–2. doi: 10.1038/s41422-018-0117-4 (PMC6318281; doi:10.1038/s41422-018-0117-4)
Supplement: Supplementary file 1 — Supplementary information, figures [file 41422_2018_117_MOESM1_ESM.pdf]

Supplementary Information

Materials and Methods

Cell culture and antibodies

HEK293T and NIH-3T3 cells were cultured in DMEM medium (Gibco) supplemented with 10% (v/v) FBS (Gibco) and 1% penicillin/streptomycin (Gibco) at 37 °C with 5% CO_2. HEK293 suspension cells were cultured in SMM 293-TI (Sino biological) supplemented with 1% FBS (Gibco) and 1% penicillin/streptomycin (Gibco) in an incubator with shaking at 130 rpm. Rabbit anti-m^6A was purchased from Synaptic Systems.

siRNA knockdown

Two duplex RNAi oligos targeting PCIF1 mRNA sequences were designed and synthesized by GenePharma (Shanghai, China). One thing should be noted that the PCIF1 gene name in NCBI database is PDX1 C terminal inhibiting factor 1, which is distinguished from mouse PDX1 C terminal interacting factor 1(PCIF1, also known as SPOP). PCIF1 siRNA with the target sequence 5′-CCCUACUACUUCAACCGAUTT-3′; PCIF1 siRNA-b with the target sequence 5′-CCUUCCAUGUUUCGUGAAATT-3′. A scrambled duplex RNAi oligo (5′-UUCUCCGAACGUGUCACGUTT-3′) was used as the negative control. HEK293T cells were transfected with 30 nM siRNA duplex two times using Lipofectamine RNAiMAX (Thermo Fisher Scientific) according to the manufacturer’s instructions and harvested 48h after the first transfection. The knockdown efficiency of PCIF1

mRNA was quantified by qRT-PCR and normalized to GAPDH mRNA. qPCR primers were listed as follows:

PCIF1-qFWD: GTCAGCCGCAACTACTTC;

PCIF1-qRVS: CACGCCGAACATCATCTG；

GAPDH-qFWD: CAACAGCCTCAAGATCATCA;

GAPDH-qRVS: AGTCCTTCCACGATACCAA；

Mouse-PCIF1-qFWD: GGCAACGGAGTGAAGAAG;

Mouse-PCIF1-qRVS: CAGCATTGGTCTGGATGTC；

Mouse-GAPDH-qFWD: TCTCCTGCGACTTCAACA;

Mouse-GAPDH-qRVS: CTGTAGCCGTATTCATTGTCATA；

RNA purification

Total RNA was extracted with TRIzol (Life technologies) followed by isopropanol precipitation, according to the manufacturer’s instructions (Invitrogen). PolyA+ RNA was purified from total RNA with two sequential rounds of polyA tail purification using oligo(dT)$_{25}$ Dynabeads (Invitrogen).

Synthesis of oligonucleotides

The oligoes with 5' cap structure were synthesized on an ABI Expedite 8909 DNA/RNA synthesizer (Applied Biosystems). Standard RNA phosphoramidites and 3'-BiotinTEG CPG were purchased from Glen research. RNA sequences were prepared at 1 μmol scale using standard protocols for solid-phase RNA synthesis. The last coupling step was followed by a DMT-off procedure and the following

synthesis of ppp-RNAs were achieved on solid support as previously described[1]. Oligonucleotides were released from the solid support, deprotected, and desalted by Sep-Pak C18 Plus Short Cartridge (Waters). Finally, all RNA sequences were capped using Vaccinia Capping System (M2080S, NEB) at 37 °C for 2 h to obtain the m^7Gppp-RNA. LCQ Fleet linear ion trap mass spectrometry (Thermo Fisher Scientific) was used to confirm the mass of RNA oligonucleotides produced. Probe-2 with internal Am was synthesized by Takara (Dalian, China). Probe sequences were listed as follows:

Probe-1 from vaccinia virus mRNA:

5′-AmCAUAGCAGGCAUGGAAUCCUUCAA-biotin-3′

Probe-2 from vaccinia virus mRNA:

5′-ACAUAGCAmGGCAUGGAAUCCUUCAA-3′

Probe-3 from human mRNA:

5′-AmCCCACUCCCGCUGCCCCGUCCGGCC-biotin-3′

Probe-4 from human mRNA:

5′-ACCCACUCCCGCUGCCCCGUCCGGCC-biotin-3′

In vitro methylation assay

A standard reaction mixture contained 15 mM HEPES pH7.9, 10 mM DTT, 1 mM SAM (NEB), 1 U/μl SUPERaseIn RNase Inhibitor (Invitrogen), 100ng purified protein or 10 μl partially purified fractions and 1 μg RNA probe in a total volume of 40 μl. Prior to the reaction, the RNA probes were denatured at 65 °C for 5 min and chilled

on ice immediately. The reaction mixtures were then incubated at 37 °C for 2 h. For methylation assay of recombinant protein, RNA was directly purified by phenol chloroform extraction followed by ethanol precipitation. For methylation assay of cell extract or partially purified fractions, RNA was firstly extracted using miRNeasy Mini Kit (QIAGEN), according to the manufacturer's instruction, and eluted with 60 µl nuclease-free H_2O. RNA was then enriched by pulling down with Streptavidin C1 dynabeads (Life Technologies). 30 µl C1 beads were washed and resuspended in 200 µl Binding buffer (0.5% SSC, 1% SDS). The 60 µl RNA was added into resuspended beads and incubated at room temperature for 1 h with gentle rotation, followed by four times of washing with Wash buffer (10 mM Tris pH 7.5, 5 mM EDTA, 1% SDS). Enriched RNA was eluted at 95 °C for 3 min using Wash buffer, then purified by ethanol precipitation. RNA pellet was dissolved in 10 µl nuclease-free H_2O and digested into single nucleosides for quantification of m^6Am level by LC-MS/MS.

Quantification of m^6A and m^6Am level by LC-MS/MS

150 ng isolated mRNA or synthetic oligonucleotides were decapped with 10 units of RppH (NEB) in ThermoPol buffer for 3 h at 37 °C. RNA was purified by ethanol precipitation and subsequently digested into single nucleosides by 0.5 U nuclease P1 (Sigma, N8630) in 40 µl buffer containing 10mM ammonium acetate, pH 5.3 at 42 °C for 6 h, then mixed with 5 µl 0.5 M MES buffer, pH 6.5 and 0.5 U Shrimp Alkaline Phosphatase (NEB, M0371S), in a final reaction volume of 50 µl adjusted with water,

and incubated at 37 °C overnight. The nucleosides were separated by ultra-performance liquid chromatography with a ZORBAX SB-Aq column (Agilent), and then detected by triple-quadrupole mass spectrometer (AB SCIEX QTRAP 5500). A multiple reaction monitoring (MRM) mode was adopted: m/z 282.0 to 150.1 for m^6A, m/z 296.0 to 150.0 for m^6Am, m/z 268.0 to 136.0 for A, m/z 282.0 to 136.0 for Am. 5 μl of the solution was injected into LC-MS/MS. Standard curves were generated by running a concentration series of pure commercial nucleosides (Berry associated). Concentrations of nucleosides in RNA samples were calibrated by standard curves.

Plasmids, expression and purification of PCIF1

The *PCIF1* gene was amplified by PCR from a human cDNA library and cloned into the NdeI/XhoI sites of pET28a vector to create pET28-PCIF1. The PCIF1 mutant protein was made by SOEing PCR with pET28-PCIF1 as the template. The mouse *Pcif1* gene was amplified by PCR from a mouse heart cDNA library and cloned into the NdeI/EcoRI sites of pET28a vector. The primers were listed below:

PCIF1-WT-FWD: GAATTCCATATGGCCAATGAGAATCACG

PCIF1-WT-RVS: AGCCTCGAGTTAAGTGGGGTGAGGCTCG

PCIF1-N553A-FWD: TGGTTCATTTGAGGCCGCGCCTCCCTTCT

PCIF1-N553A-RVS: AGAAGGGAGGCGCGGCCTCAAATGAACCA

PCIF1-F556G-FWD: TTCATTTGAGGCCAACCCTCCCGGCTGCGAGGAGC

PCIF1-F556G-RVS: AGCTCCTCGCAGCCGGGAGGGTTGGCCTCAAATGAA

Mouse-PCIF1-FWD: GAATTCCATATGGCCAATGAGAATCACGGCAGC

Mouse-PCIF1-RVS: CCGGAATTCTTAAGTGGGGTGAGGCTCTCTAC

All recombinant expression plasmids were transformed in Rosetta (DE3) cells. Colonies were grown in LB medium at 37 °C until the OD600 was between 0.8-1, and then induced with 0.4 mM IPTG overnight at 16°C. Cells were harvested by centrifugation at 3,500 g for 15 min and resuspended in ice-cold lysis buffer (50 mM MES pH 6.5, 300 mM NaCl, 10 mM imidazole). The cells were disrupted using an Ultrasonic Homogenizer and lysate was precipitated by ultracentrifugation at 11000g for 1 h at 4 °C. Collected supernatant was loaded on HiTrap His column (GE Healthcare) and eluted with buffer containing 50 mM MES pH 6.5, 300 mM NaCl, 500 mM imidazole, fractions were then collected and buffer exchanged to the buffer containing 50 mM MES pH 6.5, 50 mM NaCl, 1mM DTT and loaded on HiTrap SP column (GE Healthcare), then eluted with buffer containing 50 mM MES pH 6.5, 1M NaCl, 1mM DTT. Purified proteins were finally obtained by Gel-filtration chromatography (HiLoad 16/600 Superdex 200 pg column, GE Healthcare) equilibrated in storage buffer containing 10 mM HEPES-NaOH pH 7.0, 100 mM NaCl and 1mM dithiothreitol (DTT). Proteins were stored at −80 °C with the addition of 10% glycerol.

Fractionation of cell extract

800 ml HEK293 suspension cells [~ 100 plates (10 cm ID)] were used to purify endogenous PCIF1, cells were harvested by centrifugation at 4 °C, washed three times with cold PBS buffer. The nuclear and cytoplasmic fractionations were

performed as described previously [2]. The cytoplasmic fractionation was lysed in buffer A (10 mM Tris pH7.4, 10 mM NaCl, 1.5 mM $MgCl_2$, 1 mM DTT). Solid $(NH_4)_2SO_4$ was added to the cytoplasmic extract to achieve 50% saturation and then the mixture was incubated at 4 °C for 4 h by gently rotating. The mixture was centrifuged at 15,000 g for 15 min, and the pellet was resuspended in 5 ml buffer B (10 mM Tris pH7.4, 1 mM EDTA, 1 mM DTT, 10% glycerol) and dialyzed 24 h against 2 L changes of buffer B. The supernatant was collected by centrifugation at 4,000 g for 5 min and loaded on HiTrap Q column (GE Healthcare) equilibrated with buffer B, eluted sequentially with 0.5 M NaCl in buffer B. Fractions were assayed with RNA probe. Active fractions were pooled, concentrated and subjected to HiTrap SP column (GE Healthcare). Active fractions from HiTrap SP column were purified by HiTrap Heparin column (GE Healthcare) equilibrated with buffer B, eluted sequentially with 0.5 M NaCl in buffer B. Active fractions were running on SDS-PAGE gel and analyzed by mass spectrometry.

Protein identification by mass spectrometry

Fractions from the last step of purification route were resolved by SDS-PAGE and visualized by coomassie blue staining. For protein identification, one lane from the fraction containing the highest methylation activity was chosen for mass spectrometry analyses. Each sample was cut out of the gel and destained with a solution of 100 mM ammonium bicarbonate in 50% acetonitrile followed by dithiothreitol reduction and iodoacetamide alkylation, the proteins were digested with

sequencing grade modified porcine trypsin (Promega) overnight at 37 °C (ref.3). The resulting tryptic peptides were sequentially extracted from the gel pieces with acetonitrile include 0.1% formic acid (FA). The samples were dried in a vacuum centrifuge concentrator at 30 °C for 4 h and resuspended in 10μl 0.1%FA. 5ul of sample were loaded at a speed of 0.3 μl/min in 0.1% FA onto a trap column (C18, Acclaim PepMap™ 100 75μm x 2cm nanoViper Thermo), The peptides were then eluted across a fritless analytical resolving column (C18, Acclaim PepMap™ 75μm x15cm nanoViper RSLC Thermo) with a 75-min gradient of 4 to 30% LC-MS buffer B containing 0.1% FA and 80% acetonitrile at 300 nl/min.

Peptides were directly injected into a Thermo Orbitrap Fusion Lumos using a nano-electrospray ion source with electrospray voltages of 2.2 kV. Full scan MS spectra were acquired in the Orbitrap mass analyzer with the resolution set to 60,000 (FWHM) at m/z 200 Da. The MS data were alignment with Mouse Reviewed Swiss-Port database by Proteome Discoverer 2.2 software.

m^{6}A-seq

This procedure was performed according to the previously described m^{6}A-seq method with several modifications [4]. RNA was isolated from HEK293T cells with PCIF1 knock-down and the negative control, respectively. Two library preparation procedures were used: Method I includes a random priming step while Method II uses a template-switch RTase that preserves the 5'-end information of immunoprecitated RNA. Combining the known fact that m^{6}Am is located at the first

transcribed nucleotide, Method II allows accurate identification of m^6Am sites. Method I used 5 μg polyA+ RNA, randomly fragmented RNA into ~100 nucleotides by magnesium RNA fragmentation buffer (New England Biolabs) and incubated with 2.5 μg m^6A-specific antibody (202003, Synaptic Systems) in m^6A IP buffer at 4 °C for 2 h. The m^6A-containing fragments were incubated with preblocked Protein A Dynabeads (Thermo Fisher Scientific) at 4 °C for 2 h, and then eluted with 6.7 mM m^6A nucleoside (Berry&Associates) in 1X IP buffer at 4 °C for 1 h followed by ethanol precipitation. Fragmented polyA+ RNA (as "input") and immunoprecipitated RNA fragments were subjected to library construction using NEBNext® Ultra™ Directional RNA Library Prep Kit for Illumina® (NEB) following manufacturer's recommendations. Method II used 3 μg total RNA, randomly fragmented RNA into ~100 nucleotides and incubated with 6 μg m^6A-specific antibody (ABE572, Millipore) in m^6A IP buffer at 4 °C for 2 h, and then eluted with 6.7 mM m^6A nucleoside (Berry&Associates) in 1X IP buffer at 4 °C for 1 h followed by ethanol precipitation. Fragmented polyA+ RNA (as "input") and immunoprecipitated RNA fragments were subjected to library construction using SMARTer Stranded Total RNA-Seq Kit (Pico Input Mammalian) from Takara. Library sequencing was performed on llumina Hiseq X10 with paired-end 2 X 150 bp read length.

Reads pre-processing and alignment

Raw sequencing reads produced from m^{6}A-seq were subjected to Trim_galore (http://www.bioinformatics.babraham.ac.uk/projects/trim_galore/) for adaptor trimming and quality control. The minimum quality threshold was set to 20, and the minimum length required for reads after trimming was 30 nt. Processed reads were mapped to human genome (hg19, UCSC Genome Browser) using tophat aligner (v2.0.13) with default parameters.

Identification of putative m^{6}Am and m^{6}A sites

Peak calling within using MACS2, the effective genome size was set to be 2.7*10e9 under the option of –*nomodel* and *p* value cutoff 0.01. Peak annotated by annotatePeaks.pl (Homer V4.8). Two parameters were defined to classify m^{6}Am and m^{6}A sites: distance to TSS site and whether include GGACH motif. A position was identified as m^{6}Am site when meeting following criteria: 1) peak was overlap with TSS site；2) without GGACH motif in peak. m^{6}A site was identified when peak was not in TSS and contain GGACH motif.

Quantification and statistical analysis

The p-values were calculated using unpaired two-sided Mann-Whitney U-test. *$P < 0.05$; **$P < 0.01$; ***$P < 0.001$; ns, not significant. Error bars represent mean ± SD.

Data and software availability

Sequencing data have been deposited into the Gene Expression Omnibus (GEO) under the accession number: GSE120229.

Reference

1 Zlatev, I. *et al.* Efficient solid-phase chemical synthesis of 5'-triphosphates of DNA, RNA, and their analogues. *Org Lett* **12**, 2190-2193 (2010).

2 Keith, J. M., Ensinger, M. J. & Mose, B. HeLa cell RNA (2'-O-methyladenosine-N6-)-methyltransferase specific for the capped 5'-end of messenger RNA. *J Biol Chem* **253**, 5033-5039 (1978).

3 Olsen, J. V., Ong, S. E. & Mann, M. Trypsin cleaves exclusively C-terminal to arginine and lysine residues. *Mol Cell Proteomics* **3**, 608-614 (2004).

4 Dominissini, D., Moshitch-Moshkovitz, S., Salmon-Divon, M., Amariglio, N. & Rechavi, G. Transcriptome-wide mapping of N(6)-methyladenosine by m(6)A-seq based on immunocapturing and massively parallel sequencing. *Nat Protoc* **8**, 176-189 (2013).

Figure S1. Purification of column fractions containing endogenous methylation activity, and the subsequent over-expression and purification of the recombinant PCIF1 protein.

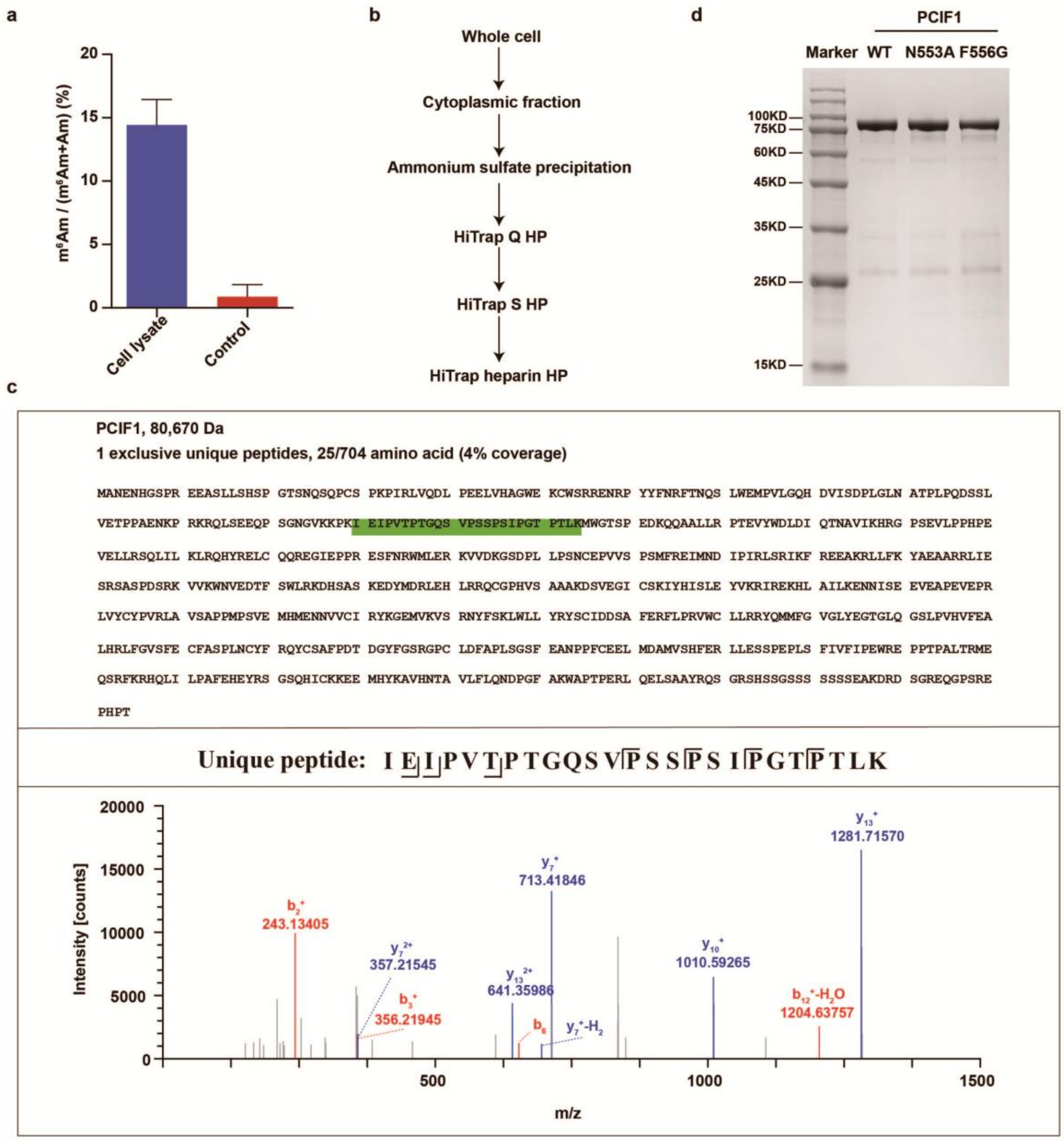

a. LC-MS/MS quantification of the m^{6}Am/(m^{6}Am+Am) ratios in probe-1 incubated with HEK293 suspension cell lysate or control (inactivated lysate) (n = 3).

b. The purification route for endogenous methylation activity from HEK293 suspension cell extract.

c. Unique peptide identification of PCIF1 by mass spectrometry, from the column fractions containing the highest endogenous methylation activity.

d. Coomassie blue staining of recombinant PCIF1 proteins.

Figure S2. Methylation activity of PCIF1 *in vivo* and *in vitro*.

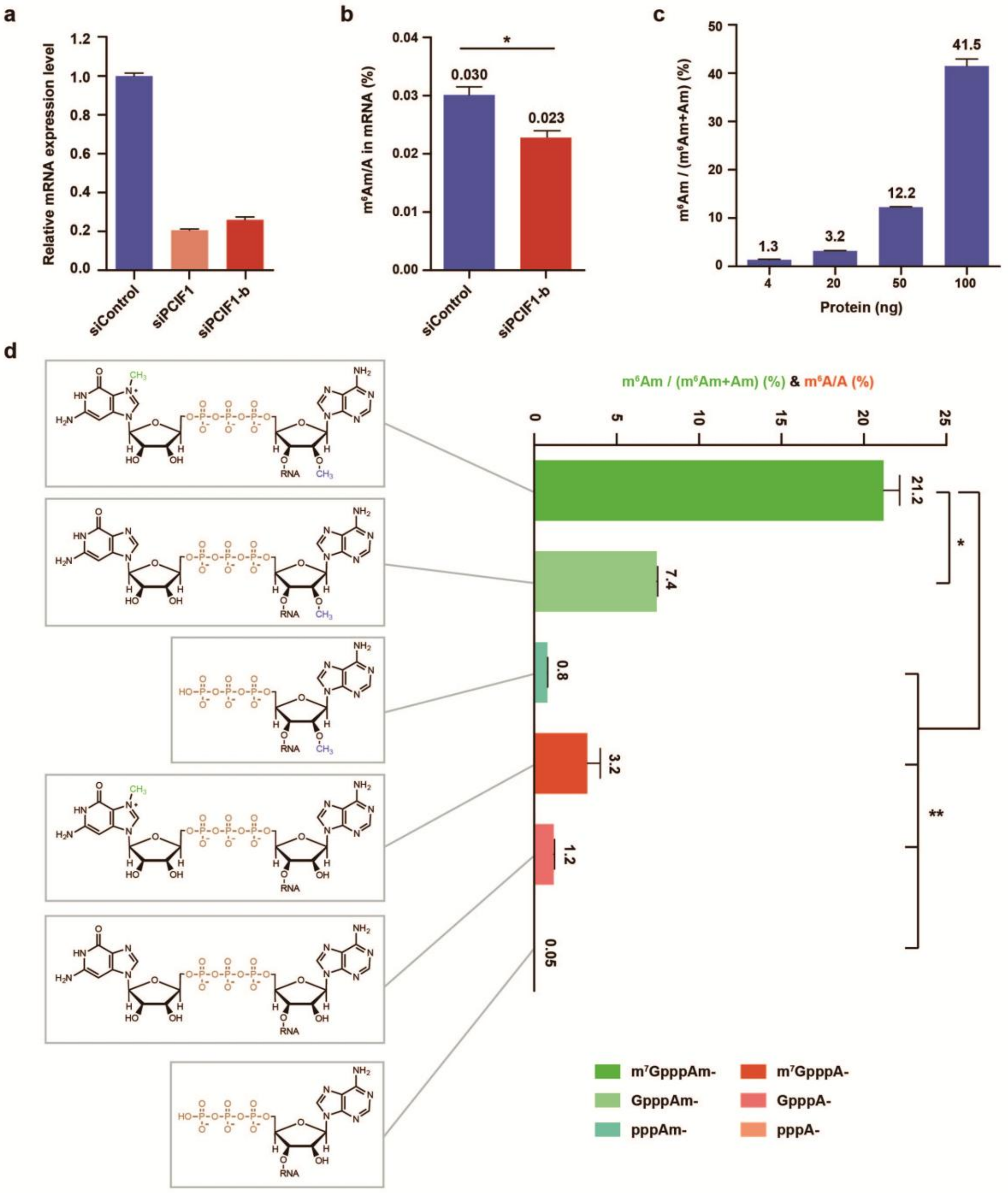

a. qRT-PCR to measure knockdown efficiency in HEK293T cells transfected with control, PCIF1 siRNA or PCIF1 siRNA-b (n = 3).

b. LC-MS/MS quantification of the m^{6}Am/A ratios in polyadenylated RNA of HEK293T cells treated control or siRNA-b (n = 3).

c. LC-MS/MS quantification of the $m^6Am/(m^6Am+Am)$ ratios in probe-1 incubated with different amounts of recombinant PCIF1 (n = 3).

d. The methylation activity of PCIF1 towards various probe-3 and probe-4 substrates. Respective chemical structures are drawn on the left (n = 3). The p value was assessed using Student's t-test, with $^*P < 0.05$; $^{**}P < 0.01$; $^{***}P < 0.001$; ns, not significant. Error bars represent mean ± SD for n=3 experiments.

Figure S3. Methylation activity of mouse PCIF1 *in vivo* and *in vitro*, and results of m⁶A-seq experiments.

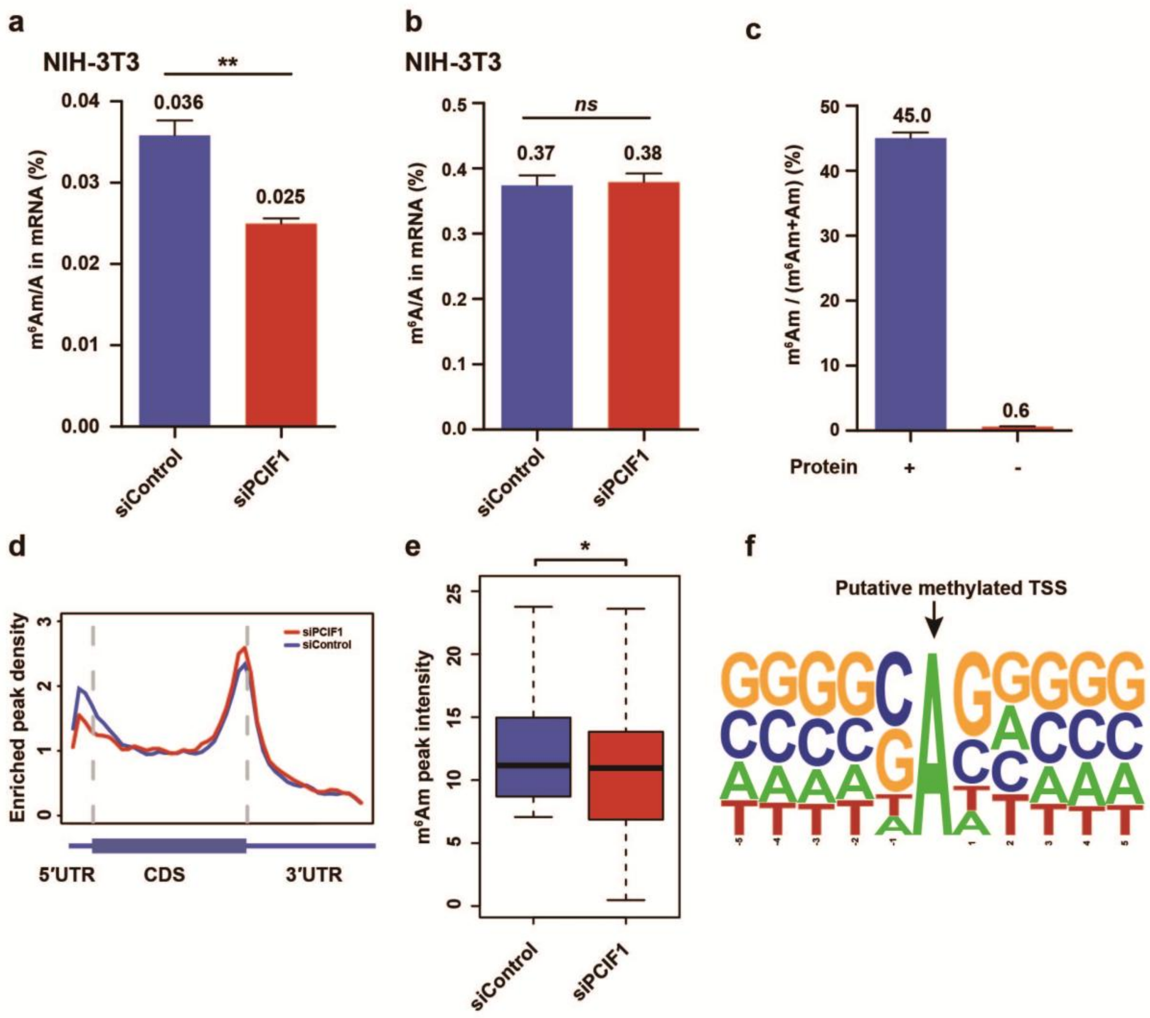

a. LC-MS/MS quantification of the m^6Am/A ratios of NIH-3T3 polyA+ RNA treated with control or *mPcif1* siRNA (n = 3).

b. LC-MS/MS quantification of the m^6A/A ratios of NIH-3T3 polyA+ RNA treated with control or *mPcif1* siRNA (n = 3).

c. LC-MS/MS quantification of the methylation activity of mouse PCIF1 protein (n = 3). The *p* value was assessed using Student's t-test, with $*P < 0.05$; $**P < 0.01$; $***P < 0.001$; ns, not significant. Error bars represent mean ± SD for n=3

experiments.

d. Distribution of enriched m^6A/m^6Am peak density across mRNA segments of control and *mPcif1* knockdown samples using m^6A-seq (Method II).

e. Boxplot of m^6Am peak intensity in PCIF1 KD and control mRNA using m^6A-seq (Method II).

f. Sequence logo for a set of high confidence m^6Am sites using m^6A-seq (Method II). The position of the putatively methylated TSS is indicated.
